# Memoir study: Investigating image memorability across developmental stages

**DOI:** 10.1371/journal.pone.0295940

**Published:** 2023-12-20

**Authors:** Gal Almog, Saeid Alavi Naeini, Yu Hu, Emma G. Duerden, Yalda Mohsenzadeh

**Affiliations:** 1 Western Institute for Neuroscience, University of Western Ontario, London, Ontario, Canada; 2 Department of Computer Science, University of Western Ontario, London, Ontario, Canada; 3 Department of Pathology and Laboratory Medicine, University of Western Ontario, London, Ontario, Canada; 4 Applied Psychology, Faculty of Education, University of Western Ontario, London, Ontario, Canada; 5 Vector Institute for Artificial Intelligence, Toronto, Ontario, Canada; Federal University of Paraiba, BRAZIL

## Abstract

Images have been shown to consistently differ in terms of their memorability in healthy adults: some images stick in one’s mind while others are forgotten quickly. Studies have suggested that memorability is an intrinsic, continuous property of a visual stimulus that can be both measured and manipulated. Memory literature suggests that important developmental changes occur throughout adolescence that have an impact on recognition memory, yet the effect that these changes have on image memorability has not yet been investigated. In the current study, we recruited adolescents ages 11–18 (n = 273, mean = 16) to an online visual memory experiment to explore the effects of developmental changes throughout adolescence on image memorability, and determine if memorability findings in adults can be generalized to the adolescent age group. We used the online experiment to calculate adolescent memorability scores for 1,000 natural images, and compared the results to the MemCat dataset—a memorability dataset that is annotated with adult memorability scores (ages 19–27). Our study finds that memorability scores in adolescents and adults are strongly and significantly correlated (Spearman’s rank correlation, *r* = 0.76, *p* < 0.001). This correlation persists even when comparing adults with developmentally different sub-groups of adolescents (ages 11–14: *r* = 0.67, *p* < 0.001; ages 15–18: *r* = 0.60, *p* < 0.001). Moreover, the rankings of image categories by mean memorability scores were identical in both adolescents and adults (including the adolescent sub-groups), indicating that broadly, certain image categories are more memorable for both adolescents and adults. Interestingly, however, adolescents experienced significantly higher false alarm rates than adults, supporting studies that show increased impulsivity and reward-seeking behaviour in adolescents. Our results reveal that the memorability of images remains consistent across individuals at different stages of development. This consistency aligns with and strengthens prior research, indicating that memorability is an intrinsic property of images. Our findings open new pathways for applying memorability studies in adolescent populations, with profound implications in fields such as education, marketing, and psychology. Our work paves the way for innovative approaches in these domains, leveraging the consistent nature of image memorability across age groups.

## Introduction

Every day, humans observe and interact with hundreds of images and scenes; whether it be on a cellphone, on television, or in print. While human memory has a massive capacity to remember images [1], some are instead forgotten, either immediately or after variable lengths of time. Memorability is indeed a property intrinsic to all images that can be extracted, as well as predicted [2–8], or manipulated [9].

Image memorability can most simply be described as the extent to which an image is remembered by human observers after a single exposure. It is defined and quantified as the probability that a repetition of a photograph will be detected by an observer when presented amidst a stream of images at various intervals [10]. Isola and colleagues [2] introduced their large-scale visual memory game used to test and quantify the memorability of images and hosted it on Amazon Mechanical Turk to allow for crowdsourcing. This experiment has since been replicated many times [3, 10, 11] and has formed the foundations of measuring image memorability. Since then, several studies have demonstrated the robustness of memorability rankings across various task settings, and delays [7, 12–16]. Memorability is a critical, intrinsic property of images as previous neuroimaging studies have shown distinct brain patterns differentiating high and low memorability images that appear unconsciously, even when participants are not performing a memory task [4, 17, 18]. Therefore, memorability cannot be overridden by an individual: one cannot attempt to forget a memorable image, or remember a forgettable image [17]. To understand memorability, the visual memory game described above has been used to assign memorability scores to thousands of images, and it has been shown that certain image features can be extracted and linked to memorability. Specific object categories (people, animals, and vehicles) as well as scene categories (indoor scenes) are more memorable than others [10–12, 19, 20]. In fact, Isola et al. [10] have reported that the scene category captures most of what makes an image memorable. They also demonstrated that high-level semantic attributes, such as spatial layout, dynamics, and location, are associated with memorability. Saliency and contrast have been shown to be important factors, meaning images with specific points of focus are more memorable [12, 21]. Interestingly, image aesthetics and perceived popularity have shown little to no correlation with memorability, despite being correlated to each other [11, 12, 19]. Negative emotions are more memorable than positive emotions, so images that evoke disgust, for example, would be remembered better than those that conjure feelings of amusement [12]. Colours and colour features such as saturation and hue have little correlation with memorability. Non-semantic image statistics, such as the number of objects in the image, are also not effective predictors of memorability on their own [10].

While extensive work has been done to study image memorability in adults, no studies exist regarding the adolescent (ages 11–18) population and the differences in image memorability across different age groups at different developmental stages. There are massive differences in the lived experiences of adults and adolescents—the objects they interact with daily, their preferences, and their exposure to the external world diverge greatly. For this reason, we expect the memorability of certain images or image categories to differ across the age groups. As well, recognition memory (the ability to recognize a previously-encountered stimulus) is an element of the visual memory game used to quantify image memorability. Recognition memory has been shown to exhibit age-related differences: previous work has demonstrated that recognition memory gradually improves with age throughout childhood and adolescence [22–26]. The continued development of neural systems including the ventral temporal cortex [27, 28], the lateral prefrontal cortex [29] and the medial temporal lobes [30] may support the improvement in recognition memory throughout adolescence. Due to these age-related differences in recognition memory, we expect there to be corresponding differences in image memorability. As such, we expect adolescents to not only have lower memorability scores overall, but also have a lower correlation with adult memorability scores than seen in adult-only studies. On the contrary, if image memorability is an intrinsic feature of visual stimuli, resistant even to developmental differences in the brain, image memorability would remain consistent between different ages. This study aims to fill this gap by assigning memorability scores derived from adolescents to images and comparing them to memorability scores derived from adults. If image memorability is truly consistent even across age groups that are known to be developmentally different, this will strengthen the current belief that memorability is in fact intrinsic to the image and independent of the memory process of an individual. We investigate the intrinsic nature of image memorability and whether conclusions from research done in adults can be extrapolated to the adolescent population.

## Materials and methods

### Participants

A total of 273 participants (143 female) aged 11–18 (mean = 16, SD = 2) years were recruited from Amazon Mechanical Turk, completing an average of six blocks. We recruited participants ages 11–18 in order to cover the span of important developmental stages associated with memory during adolescence [29–31], while ensuring participants were old enough to meaningfully participate in the experiment. Our participant inclusion criteria were as follows: individuals aged 11–18 years old who are typically developing children and have the ability to read and write in English. The exclusion criteria included any diagnosis of a learning disability, brain injury, or current use of medications.

Participants consented to their participation following the protocol approved by Western University Research Ethics Board (Approval Letter Number 115893). In detail, 18 year old participants and parents of minor participants (ages 11–17) reviewed an informed letter of consent, and consented to participate electronically by choosing a checkbox stating “I have read the Letter of Information and consent to participate in this study”. After the parent consented, they were asked if they would like their child (ages 11–17) to participate in the study. If they said yes, they were given an assent form and asked to go through it with their child. The assent form had a checkbox that states, “I have read the Assent Letter and consent to participate in this study”. They consented by choosing the checkbox. Those individuals who were 18 years of age only went through the informed consent process. Participants were compensated for their participation in the study according to the number of blocks they completed. Participants for this study were were recruited throughout March to August 2021. The authors did not have access to any identification information.

Detailed information about participants of MemCat dataset can be found in the original MemCat paper [11]. In short, participants included 249 psychology students whose ages ranged 18 to 27 years old (mean = 19.24, SD = 0.94), and 2162 AMT-workers with reported ages ranging 18 to 82 years old (mean = 37, SD = 11.89).

### Stimuli

The stimuli in this study were selected from MemCat [11], a recently published image memorability dataset. The MemCat dataset is publicly available and includes 10,000 images that have been annotated with adult memorability scores. We used adult memorability scores provided by MemCat as a point of comparison to the adolescent memorability scores we obtained in this study. A special feature of the MemCat dataset (compared to other image memorability datasets [3, 6, 7, 10, 32]) is its hierarchical category structure: it features five main categories (animals, food, landscapes, sports, and vehicles), each with at least 20 subcategories (such as sheep, salad, waterfall, basketball, and airplane). This allowed us to robustly analyze the effects of these categories on memorability, as done previously in adults [10–12, 19, 20]. For our adolescent memorability dataset, which we call the Memoir dataset, we randomly sampled 1,000 images from the MemCat dataset equally from each category and from the full range of memorability scores provided in MemCat. Each of the selected images was reviewed by the authors and an ethics board to make sure they were appropriate for the target population (adolescents). Specifically, the images were manually and individually reviewed by three of the authors to exclude any picture with disturbing or emotionally inappropriate content. For this, images were partitioned into three subsets; each author reviewed one of the subsets and flagged any images which were potentially disturbing and inappropriate. The flagged images were reviewed and removed from the dataset subject to unanimous agreement.

### Experiment design

**Fig 1:**
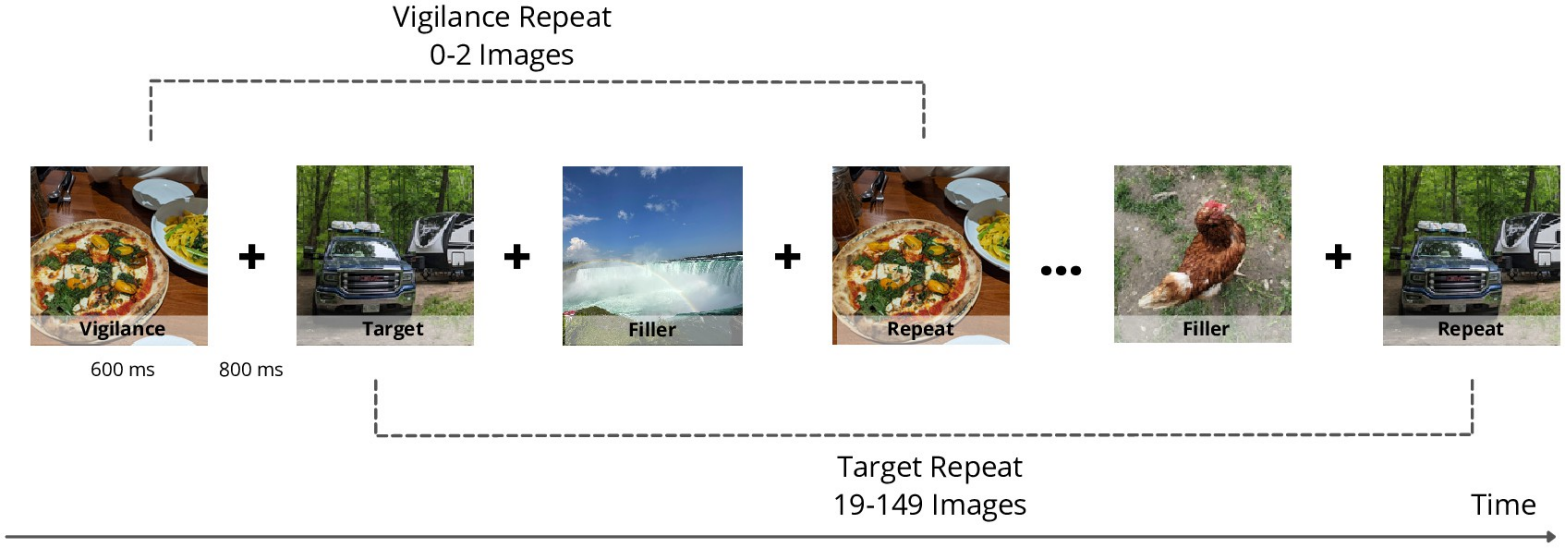
Design of experiment: Images were displayed for 600 ms followed by an 800 ms blank screen with a fixation cross. Participants were instructed to press the spacebar when they recognized a second occurrence of any image. The depicted images are examplary images and are not from our dataset.

To measure image memorability, we conducted an online visual memory game and recruited participants through Amazon Mechanical Turk. Following previous memorability work [3, 7, 10, 11, 33], the visual memory game was a repeat detection task (see schematic in Fig 1). Participants were shown a sequence of images, each displayed for 600 ms with 800 ms inter-stimulus intervals, and were instructed to press the spacebar when they encountered an image that they remembered being shown previously. The inter-stimulus intervals consisted of a blank white screen with a centered black fixation cross and were shown for 800 ms. While images may have different aspect ratios, they were presented on the screen with the same height. Participants received feedback on their performance during the experiment. If participants correctly identified a target image repeat, a green border appeared around the image, whereas in the case of a false alarm, a red border appeared around the image. The purpose of the feedback was to increase engagement of participants with the experiment and make sure that they understood the task clearly. At the start of the experiment, participants were shown a short demo video to ensure they understood the instructions.

The experiment comprised of 10 blocks, each lasting around 5 minutes. The experiment design is such that each block is independent of other blocks (each target image and corresponding target repeat were always in the same block). Participants were instructed to complete as many blocks as they wish to. Participants could complete a maximum of 10 blocks or exit the game at any time. Participants completed an average of 6 blocks each, resulting in an average of 30 minutes of active experiment time. Each block included 200 images comprised of 66 targets (and 66 target repeats), 56 fillers, and 12 vigilance repeats. Target images were repeated exactly one time and at variable time intervals (after 19–149 filler images). The filler images comprised everything in between the targets and were only displayed once, with occasional vigilance repeats of fillers being shown to ensure participants were alert [2]. Vigilance repeats were used to ensure that the participants were paying attention and participating meaningfully, and were repeated after 0 to 2 images. Both filler and vigilance images were also randomly sampled from the MemCat dataset (as were the target images). This ensured high consistency and similar types of images in the three categories (target, filler, and vigilance images), so participants were not able to differentiate between them. The fillers and vigilance repeats were the same across participants, but not necessarily in the same order—they were randomly distributed across levels of the experiment. This experiment allowed for the calculation of image memorability scores as the percentage of correct detections by participants (see Computing Memorability Scores).

Strict criteria were also used to ensure participants were engaged in the game to guarantee a high quality of results. Following [2], the visual memory game automatically ended when a participant fell below a 50% success rate on the block’s vigilance repeat detection or above a 50% error rate on the non-repeat images (“false alarms”). If this happened, all data collected from the participant from the current block was discarded. As well, if a participant did not complete a block due to quitting the experiment willingly, all data from that block was also discarded. After quality exclusions, an image’s number of responses (*N*_*resp*_) was 94, on average (range 63–164).

### Computing memorability scores

Following MemCat, we computed two different memorability scores for each image:

$$\begin{array}{c} \frac{H}{N_{resp}} \end{array}$$ (1)

$$\begin{array}{c} \frac{H-F}{N_{resp}} \end{array}$$ (2)

where *H* is the number of participants recognizing the image, *F* is the number of participants making a false alarm (ie., pressing the button when the image is presented for the first time), and *N*_*resp*_ is the total number of participants having been presented with the image.

Measure 1 is the rate of participants identifying that image repeat correctly (hit rate). Measure 2 the hit rate corrected for false alarms [34]. This is following Khosla et al. [35], who proposed that accounting for false alarms (button press when an image is shown for the first time) in the memorability score calculation reduced the influence of familiarity and the noise in the data, leading to more reliable results.

## Results

### Adolescent memorability scores

**Fig 2:**
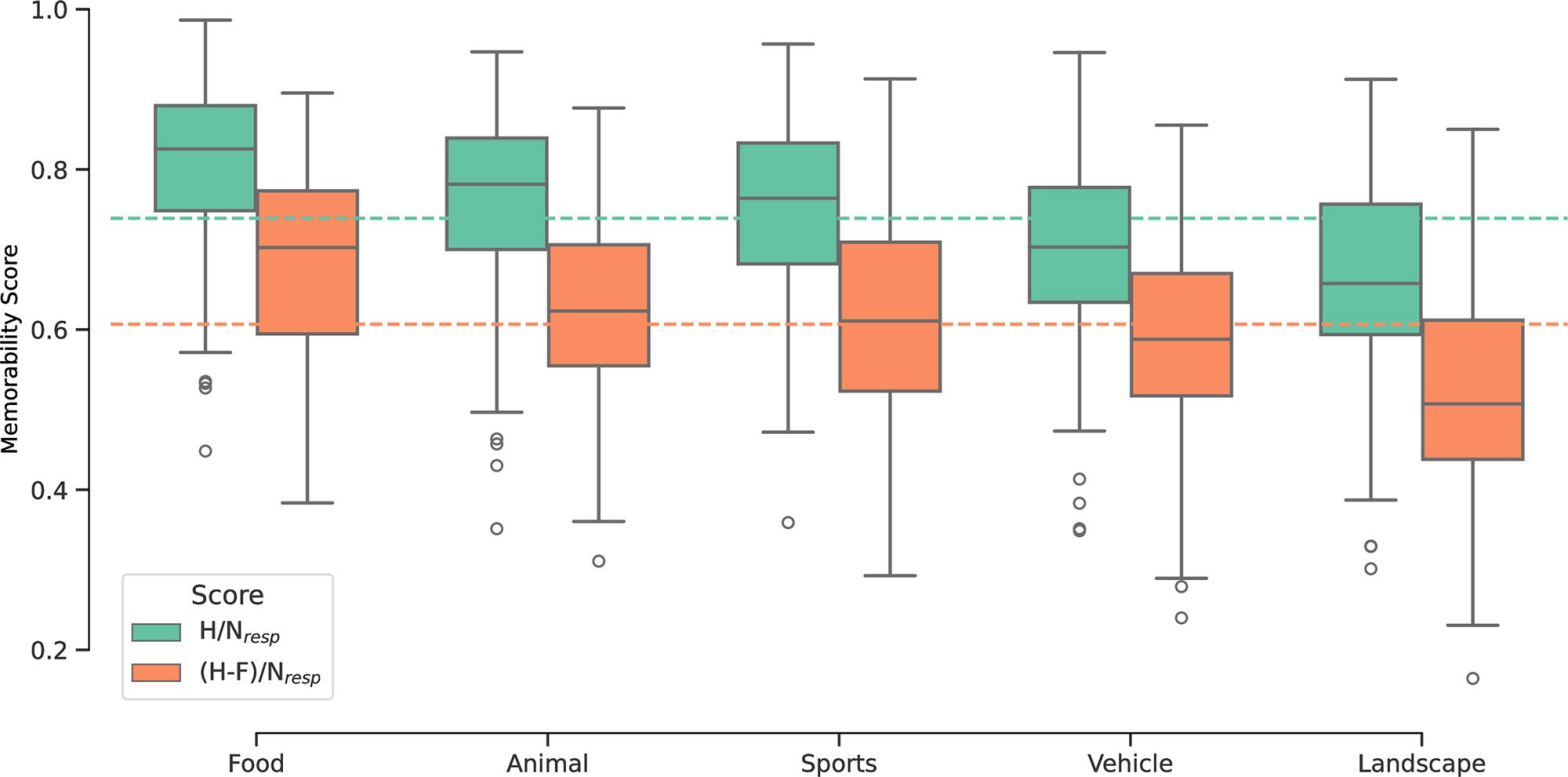
Distribution of collected memorability scores in adolescents. Green is the distribution with memorability scores computed as the hit rate across participants (*H*/*N*_*resp*_ where *H* denotes the number of participants recognizing the image, and *N*_*resp*_ is the total number of participants having been presented with the image). Orange is the distribution with memorability scores computed as the hit rate across participants and accounting for false alarms ((*H* − *F*)/*N*_*resp*_ where *F* is the number of participants making a false alarm (ie., pressing the button when the image is presented for the first time)). The horizontal lines are the global mean memorability scores in adolescents using each of the memorability calculations (*M* = 0.74 and *M* = 0.61 for *H*/*N*_*resp*_ and (*H* − *F*)/*N*_*resp*_, respectively). Number of images is *n* = 1, 000 for both figures.

Fig 2 compares the distributions of memorability scores in adolescents calculated per the two distinct memorability measures (hit rates and corrected hit rates). Memorability scores were higher when calculated without the false alarm correction ($M_{H/N_{resp}}=0.74$; $M_{\left(H-F\right)/N_{resp}}=0.61$). One-way between-subjects ANOVA revealed that there was a significant difference in memorability scores between the five main categories using both memorability measures (*α*= 0.05) [(*H*/*N*_*resp*_: *F*(4, 999) = 49.91, *p* < 0.001] [(*H* − *F*)/*N*_*resp*_: *F*(4, 999) = 48.19, *p* < 0.001]. Consistent with previous research in adults [11], images in the food category were the most memorable, followed by animal, then sports, vehicle, and finally, landscape. Table 1 summarizes the mean memorability scores in each category from both measures as well as their respective standard deviations.

**Table 1:** Mean(SD) memorability scores per category with and without false alarm correction. Number of images is *n* (all categories) = 1, 000. *n* (animal) = 195, *n* (sport) = 242, *n* (food) = 209, *n* (vehicle) = 162, *n* (landscape) = 192. The last column shows corresponding values reported in [11] for the adult population (hit rates).

Category	$M_{H/N_{resp}}$	$M_{\left(H-F\right)/N_{resp}}$	$M_{MemCat,H/N_{resp}}$
All	0.74(0.12)	0.61(0.13)	0.78
Food	0.80(0.10)	0.68(0.11)	0.59
Animal	0.76(0.11)	0.63(0.11)	0.67
Sports	0.75(0.11)	0.61(0.12)	0.60
Vehicle	0.70(0.11)	0.59(0.12)	0.64
Landscape	0.66(0.12)	0.52(0.12)	0.77

The significant and consistent trends in memorability by category suggested that broad image category (here the five main categories) explains a large amount of the variance in adolescent memorability scores. The ANOVA effect size (*η*^2^) revealed that 44% of the variance in *H*/*N*_*resp*_ memorability scores is explained by image subcategory, as well as 49% of the variance in the (*H* − *F*)/*N*_*resp*_ memorability scores. However, there is also a rather large degree of within category variation (see Fig 2) that is consistent across the five categories. Therefore, other factors may exist that contribute to the differences in image memorability scores.

### Reliability of adolescent memorability scores

**Fig 3:**
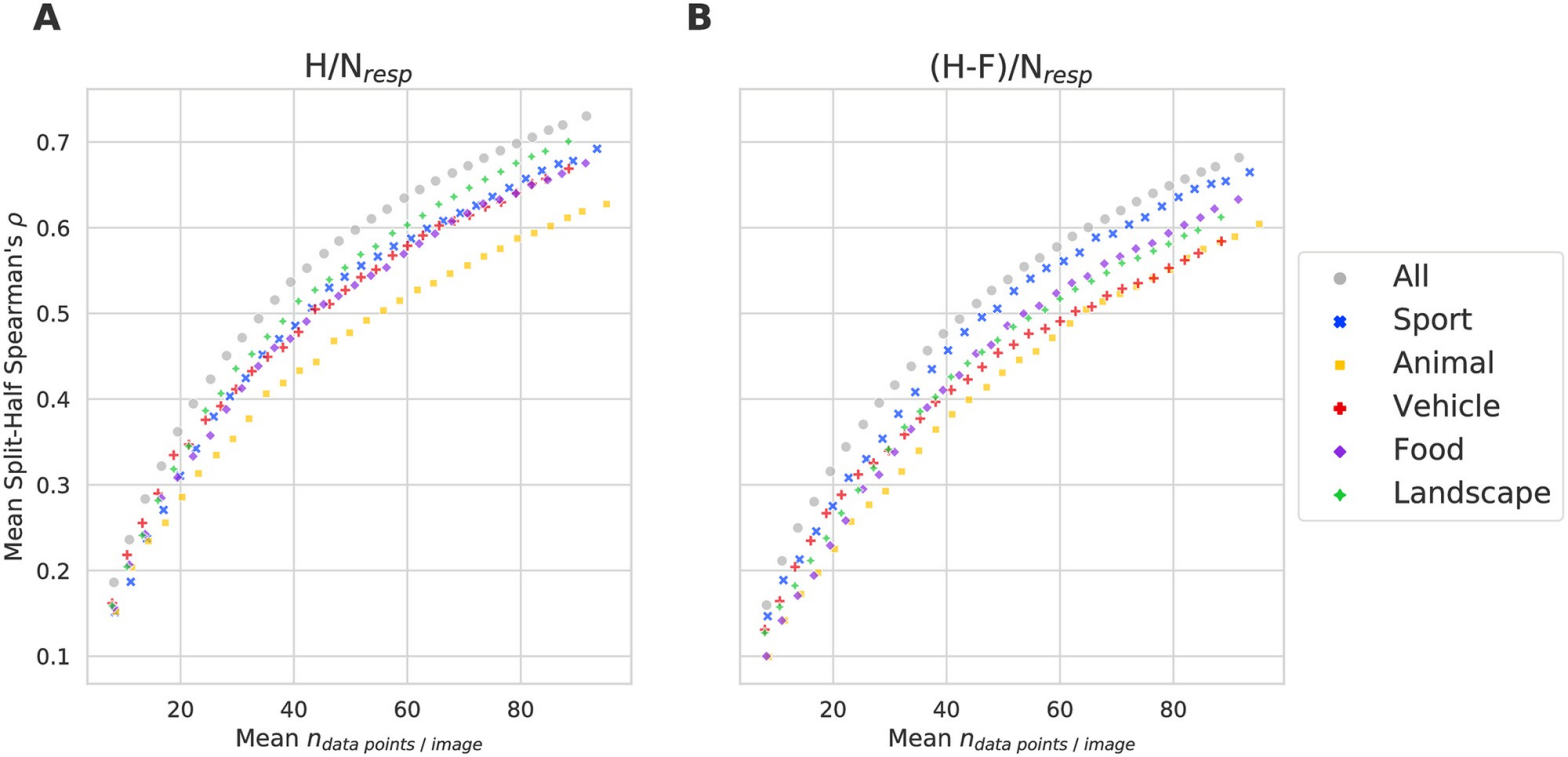
Split-half consistency across participants of the *H*/*N*_*resp*_ and (*H* − *F*)/*N*_*resp*_ memorability scores as a function of *N*_*resp*_. Estimates are based on 25 random splits. *N*_*resp*_ refers to the total number of data points per image (not to the number that goes into one half during the split-half procedure).

**Fig 4:**
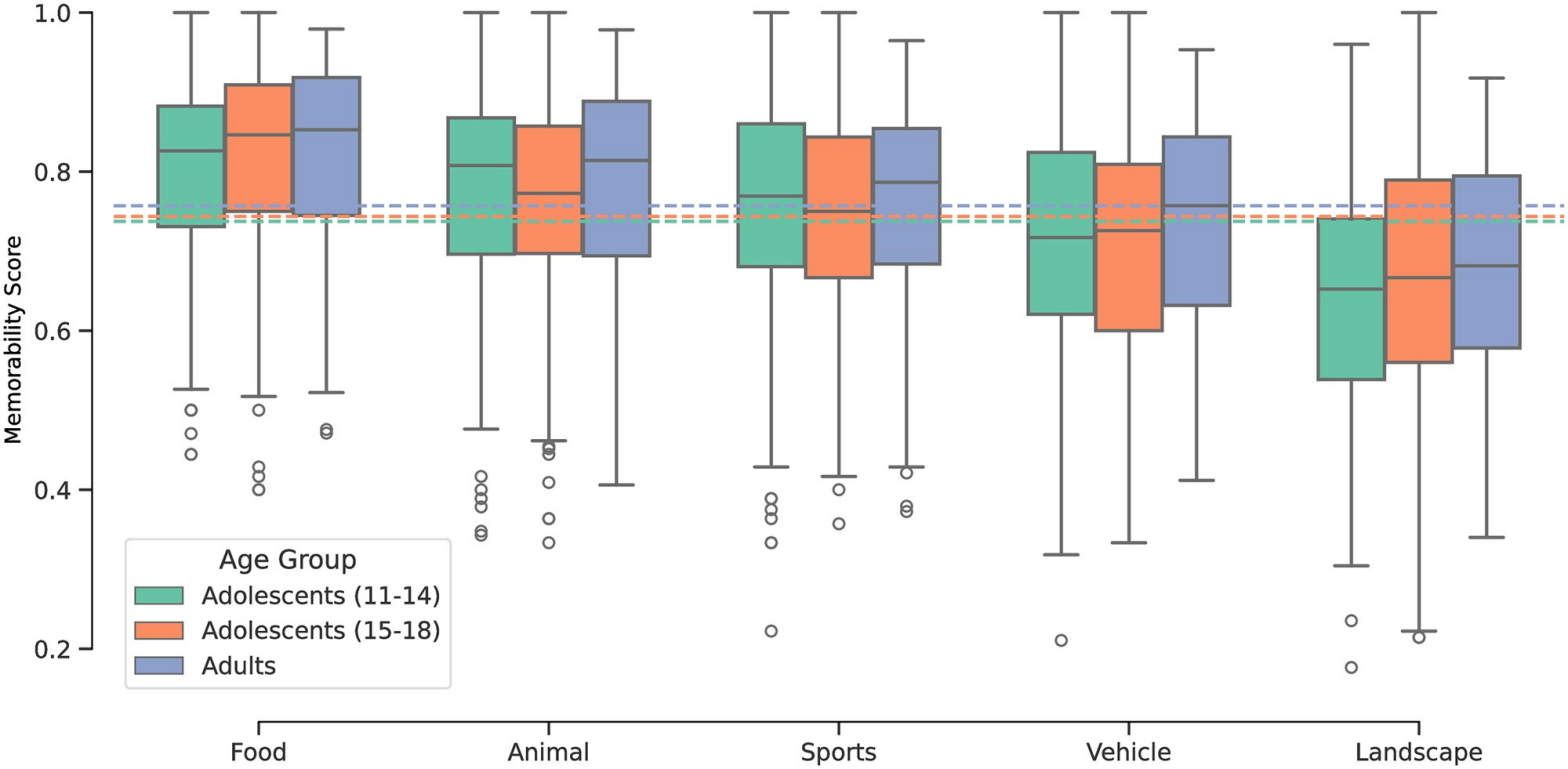
Distribution of collected memorability scores in younger adolescents (A), older adolescents (B), and adults (C), per the *H*/*N*_*resp*_ measure. Adult data is from the MemCat dataset [11]. The dashed horizontal lines represent the global mean memorability score for each of the populations (*M* = 0.74, *M* = 0.74, and *M* = 0.76 for younger adolescents, older adolescents, and adults, respectively). *n* = 1, 000 for all figures. See S1 Fig for an equivalent analysis for the (*H* − *F*)/*N*_*resp*_ scores.

Following previous memorability work [3, 10, 11], we examined the reliability of the calculated memorability scores to determine if these factors were consistent across participants. We measured the consistency and reliability of the computed memorability scores by splitting the participants into two independent sets, computing their respective memorability scores separately for each set, and calculating the Spearman’s rank correlation between the two sets (see Fig 3). To better understand the effect of the number of participants on the correlation, we also randomly down-sampled our data to a lower number of data points per image. We repeated each analysis for 25 random splits of the participant pool and averaged the Spearman’s rank correlation across the splits. Fig 3 displays the results of this analysis using both memorability measures. There is a high degree of consistency across participants both within categories and for the data as a whole, although, the consistency was slightly lower when observing individual categories (likely due to a reduced range [36]) A comparison of Fig 4 and S6 Fig reveals that the range of memorability scores is indeed smaller within the individual categories, justifying the reduced correlations. Generally, the rank correlations were higher in the *H*/*N*_*resp*_ scores. Using all available data points and across all categories, the mean split-half rank correlation using this measure was 0.73 (with a mean *N*_*resp*_ of 94). This is comparable to MemCat, who reported an overall reliability of 0.78 (with a mean *N*_*resp*_ of 99) [11]. Correlations were slightly lower within the categories: 0.70 (landscape), 0.69 (sport), 0.68 (food), 0.67 (vehicle), and 0.63 (animal). The split-half rank correlation typically underestimates the reliability that a full-length test would indicate, as reliability generally increases with the addition of more data points. Therefore, we used the Spearman-Brown formula [37, 38] to adjust these split-half estimates to reflect total reliabilities more accurately. Using this formula, we achieved the following memorability score reliabilities: 0.84 (all), 0.82 (landscape), 0.82 (sport), 0.81 (food), 0.80 (vehicle), and 0.77 (animal).

The (*H* − *F*)/*N*_*resp*_ memorability scores tended to be lower, as expected due to the nature of their calculation. Using all available data points and across all categories, the mean split-half rank correlation using this measure was 0.68 (with a mean *N*_*resp*_ of 94). Applying the Spearman-Brown formula to estimate the memorability score reliability from the split-halves, the total reliability was 0.81. In the individual categories, this was once again lower: 0.80 (sports), 0.78 (food), 0.76 (landscape), 0.75 (animal), and 0.74 (vehicle).

In sum, the strength of these correlations indicates the high reliability of measured memorability scores across the adolescent population, in spite of individual differences and other sources of noise.

### Comparing adolescent and adult image memorability scores

In order to examine the memorability differences throughout developmental stages, we divided the adolescent age group into two sub-groups: ages 11–14 (mean = 12, SD = 1, 33 male, 31 female) and ages 15–18 (mean = 17, SD = 1, 34 male, 30 female). The ages 15–18 sub-group was downsampled to contain the same number of participants (*n* = 64) as the younger sub-group. Due to the increased reliability of the memorability scores calculated using the *H*/*N*_*resp*_ measure, we restrict the following analysis to these scores. We attribute the higher split-half consistency from the scores without the false alarm correction to the significantly higher false alarms in adolescents. The increased frequency of false alarms induces greater stochasticity within our dataset, which slightly reduces their reliability. We discuss the (*H* − *F*)/*N*_*resp*_ scores and false alarm ratios separately in the next section, and the associated figures are available in the supplementary material.

**Fig 5:**
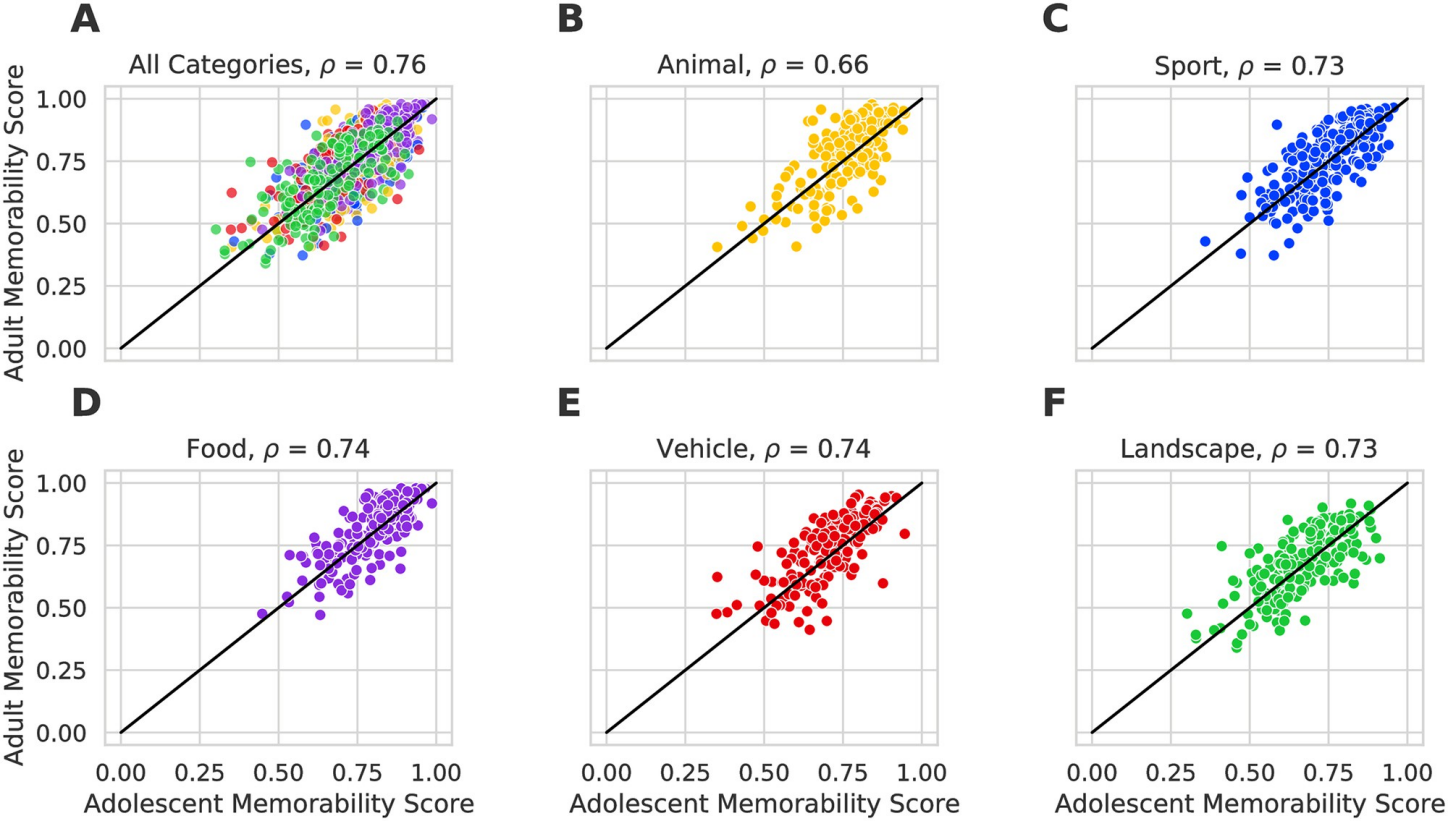
Memorability scores per image in adolescent and adult populations, per the *H*/*N*_*resp*_ measure. Each dot represents one image. The diagonal line represents identical memorability scores in adolescents and adults. Points are equally distributed around the line for all categories. Rank correlation is highest in the Sport category (C) and lowest in the vehicle category (E). See S2 Fig for an equivalent analysis for the (*H* − *F*)/*N*_*resp*_ scores.

Fig 4 displays the distribution of *H*/*N*_*resp*_ memorability scores in both adolescent sub-groups as well as the adult group for each of the five broad image categories. It also shows the global mean memorability score for each of the populations as a horizontal line. Mean memorability scores in both adolescent sub-groups were identical (*M* = 0.74, *SD* = 0.15), and comparable to mean memorability scores in adults (*M* = 0.76, *SD* = 0.14). The memorability scores of the total adolescent group and the adult group were also strongly and significantly correlated (Spearman’s rank correlation, *r* = 0.76, *p* < 0.001). We note that this rank correlation is very close to the total mean split-half rank correlation (0.84) established in (Adolescent Memorability Scores). To account for the differences in the number of responses per image in Memoir and MemCat, we used an additional down-sampling procedure to match the number of responses per image in both groups and computed the correlations. This consisted of down-sampling the responses of each target image to have an equal number of responses in both age groups, and calculating the Spearman’s rank correlation at different down-sampling levels. The down-sampled memorability scores were similarly correlated (Spearman’s rank correlation, *r* = 0.73, *p* < 0.001), so the slight differences in number of responses per image in the two groups does not have a significant effect on our results. The full results of this analysis are provided in S3 Fig. The rank correlations between the adolescent sub-groups and the adult group were also strong and significant: *r* = 0.67, *p* < 0.001 in the younger group and *r* = 0.60, *p* < 0.001 in the older group. Rank correlation between the two adolescent sub-groups was *r* = 0.58, *p* < 0.001. Note that these correlations are lower because the range of memorability scores in each sub-group is smaller than in the total adolescent population, and correlation typically depends on the range of measures being assessed [36]. These results suggest that memorability rankings are consistent not only across adolescent and adult age groups, but also within the different adolescent sub-groups that contain participants at different developmental stages. As evident in Fig 4, the category rankings are also identical in the three groups. In all age groups, the food category is the most memorable, followed by animal, sports, vehicle, and landscape. Fig 5 visualizes the rank correlation between the overall adolescent age group and the adult group, and how the memorability scores of each respective image differs between them. Points are generally evenly distributed around the equality line and exhibit a strong positive correlation. The rank correlations were lower within the categories (as expected due to the lower number of data points): 0.73 (vehicle), 0.73 (food), 0.72 (sport), 0.71 (landscape), and 0.67 (animal).

### Comparing adolescent and adult false alarm rates

**Fig 6:**
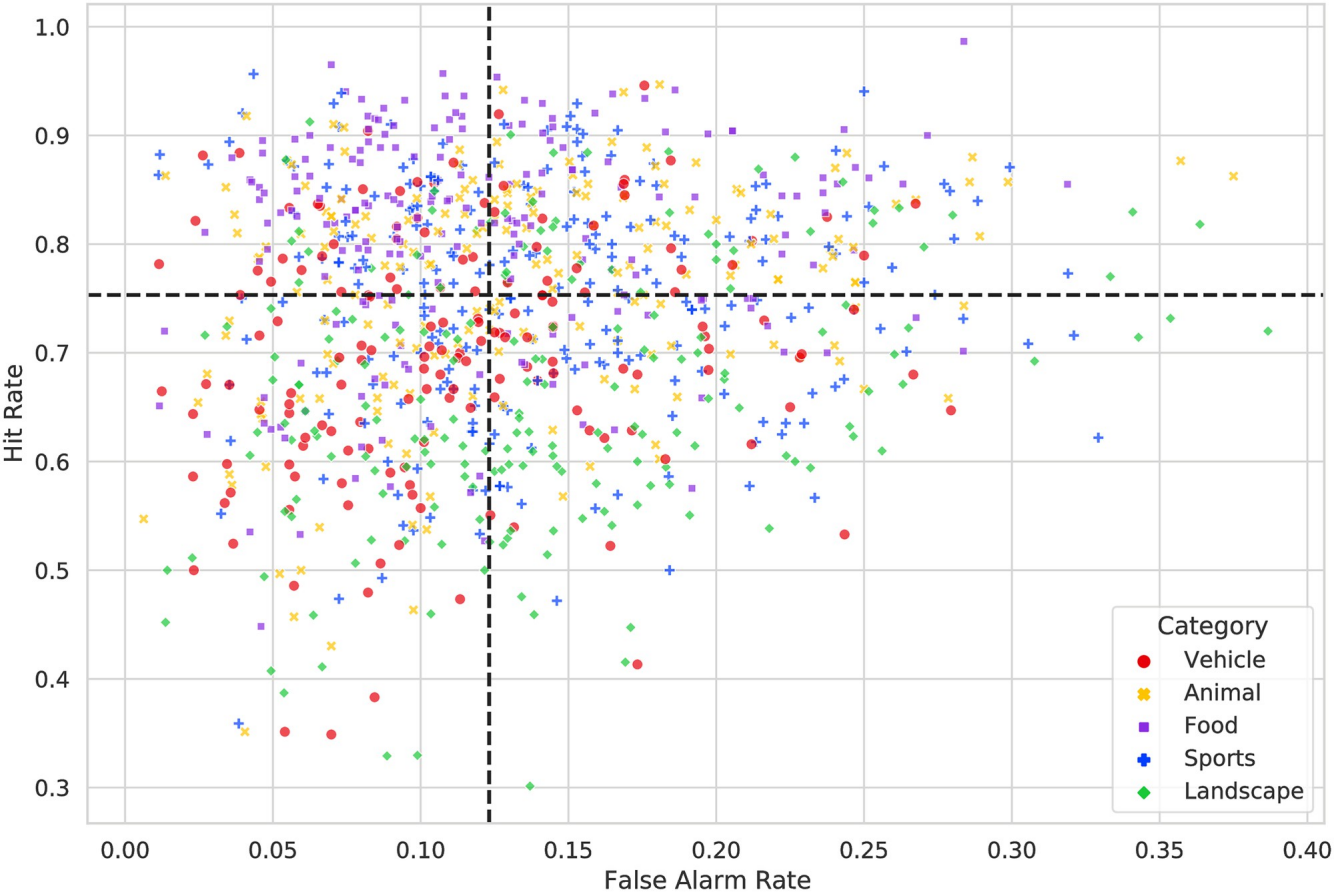
Scatter plot of hit rates (*H*/*N*_*resp*_) and false alarms rates (*F*/*N*_*resp*_) in adolescents. Black dashed lines represent the median values that divide the data into four quadrants of high/low HR/FAR.

Previous work [32] has suggested that images with a high hit rate yet a low false alarm rate are “truly memorable”, while those that receive both a high hit rate and a high false alarm rate may simply conjure strong false memories. We aimed to determine whether similar images in adolescents and adults were truly memorable. To isolate these truly memorable images, we divided the images into four quadrants consisting of high/low hit rates (*HR* = *H*/*N*_*resp*_) and false alarm rates (*FAR* = *F*/*N*_*resp*_). Following [32], these quadrants were formed by splitting the images along the median HR and FAR of the adolescent data (Fig 6).

We first note that false alarm rates, in general, were significantly higher in adolescents (*M* = 0.13, *SD* = 0.07) than in adults (*M* = 0.06, *SD* = 0.05); *t*(1, 000) = −42.81, *p* < 0.001. This is consistent with previous research in both working memory and recognition memory [39]. The increased stochasticity that is introduced with the higher number of false alarms might also explain why, in the previous section (Comparing Adolescent and Adult Image Memorability Scores), we observed a greater reduction in adolescent memorability scores and rank correlations than MemCat when using the memorability measure with the false alarm correction (see S1 Fig). However, the Spearman’s rank correlation between false alarm rates in adolescents and adults was significant (*r* = 0.55, *p* < 0.001). We compared the quadrant assignment agreements between adolescents and adults to determine if the same images were truly memorable for both populations. The truly memorable images (high HR, low FAR) had the highest percentage agreement of 62%, compared to a chance 25%. Memorable and familiar images (high HR and FAR) had an agreement of 55%, truly forgettable images (low HR and FAR) had an agreement of 59%, and the images which are commonly falsely remembered but rarely actually remembered (low HR, high FAR) had an agreement of 53%. Therefore, images are “truly memorable” to both adolescents and adults with a high degree of consistency.

As mentioned in the previous section (Comparing Adolescent and Adult Image Memorability Scores), the overall Spearman’s rank correlation between the adolescent and adult memorability scores was 0.76. Table 2 summarizes the Spearman’s rank correlations between adolescent and adult memorability scores in each of the quadrants. The truly memorable images (high HR, low FAR) had a low correlation compared to the other quadrants (*r* = 0.49) without the false alarm correction, but the strongest correlation of the four quadrants (*r* = 0.56) with the false alarm correction. The rank correlations were lower here because the sample size in each quadrant was lower than when the rank correlation was calculated using all available data. The false alarm correction increased the rank correlation of the truly memorable images, but decreased that of the truly forgettable images. Applying the correction was effective in reducing the noise in the truly memorable images, but slightly reduced our overall rank correlation and reliability (as seen in the previous sections).

**Table 2:** Spearman’s rank correlation between adolescent and adult memorability scores per the two measures. Correlations are shown separately for each HR/FAR quadrant, created by taking the median of each in the adolescent data.

Quadrant	*H*/*N*_*resp*_ *r*	(*H* − *F*)/*N*_*resp*_ *r*
High HR, Low FAR	0.49	0.56
High HR, High FAR	0.39	0.39
Low HR, Low FAR	0.61	0.50
Low HR, High FAR	0.54	0.55

## Discussion

In this work, we investigated if image memorability is consistent across viewers of different developmental stages: young adolescents (11–14 years old), older adolescents (15–18 years old), and adults. For this, we put together the Memoir dataset, including 1,000 images with their corresponding memorability scores obtained from an online memory task performed by adolescents (11–18 years old). The images were randomly sampled from the MemCat dataset [11], a large category-based memorability dataset in adults. Therefore, similar to MemCat, our dataset is also organized in five broad categories of food, animal, sports, vehicle, and landscape. We found memorability rankings were consistent across adolescents age groups (11–14, and 15–18 years old) and adults, indicating that viewer age is not a factor in determining the memorability of images. This is consistent with previous findings that show memorability is intrinsic to the image, more so than the observer [3, 10, 17, 32, 40]. There of course do exist inter-individual effects, but they are neutralized as the memorability rankings converge to their true value. In addition to individual image rankings, category rankings are also consistent across adolescents and adults. Food images are the most memorable, while landscape images are the least memorable.

Beyond the similar memorability rankings, our data also showed very similar mean memorability scores in adolescents (*M* = 0.74) and adults (*M* = 0.76). This is surprising because recognition memory gradually improves with age throughout adolescence [41, 42]; we expected to see a difference in mean scores reflected both in our comparison of raw memorability scores within adolescent sub-groups and between adolescents and adults. This finding strengthens the current literature that suggests image memorability is independent of subjective memory processes in the brain, and instead, arises as an inherent attribute of the stimulus (image). Further research should further investigate image memorability in even younger children to challenge this theory further and observe image memorability in children whose recognition memory is even less developed. Guo and Bainbridge in a very recent work [43] observed image memorability in even younger children, and found that adult-like sensitivity to image memorability emerges by the age of four through experience. This further supports our findings that adolescents indeed portray similar memorability patterns as adults across images.

Equally interesting is the recent work in older adults on the effects of cognitive decline on image memorability. A recent study [14] explored image memorability in individuals with memory impairments (such as mild cognitive impairment or subjective cognitive decline) to determine if differences in memorability on specific images could be used to distinguish between memory-impaired individuals and healthy controls. They were able to identify a set of images that were highly memorable to healthy controls but highly forgettable to individuals with memory impairments. Remarkably, some images remained consistently and highly memorable in both healthy controls and memory-impaired individuals, and memorability scores for those images could be predicted by deep learning models.

While the ranking of image memorability was similar in adolescents and adults, adolescents experienced significantly higher false alarm rates with a distinct distribution pattern. This differs from studies examining only adults [3, 32], which found that the false alarm correction improved the prediction rank correlation within their data. It is possible that when comparing memorability scores broadly from two different distributions, that the false alarm correction introduces too much variability in the data. In this study we decided to make use of the simpler *H*/*N*_*resp*_ memorability score to analyze the data as a whole, and conducted a separate false alarm rate analysis.

Adolescence is widely recognized as a developmental period during which risk-taking and reward-seeking behaviours are increased. By the time they reach adulthood, adolescents’ risk-taking declines due to structural and functional changes in the brain’s cognitive control system [44, 45]. The increased cognitive control that comes with the maturation of the brain [46] may explain the higher false alarm rates we observed in adolescents. Their increased impulsivity and need for immediate rewards combined with the rapid nature of the visual memory game may have caused adolescents to be less patient or cautious with their button presses than adults. This is also consistent with a recent work by [39] which shows higher false alarm rate in teens than young adults (in twenties) when tested on working memory and memory recognition tasks. The authors find a lower false alarm rate in a 0-back task compared to a 2-back task. This indicates a correlation between the task load and the false alarm rates in adolescents. In our experiment target images were repeated after 19–149 filler images; this makes our task more challenging and hence an increase in the false alarm rates is expected. Despite this, we find that for both adolescents and adults the same images are likely to fall into the “truly memorable” category. A Bland-Altman analysis of the Memoir and MemCat data, using both the scores with the false alarms and without, revealed that the mean difference of memorability scores is higher using the false alarm correction (there are more discrepancies). Further, we observed no relationship between the Memoir and MemCat discrepancies and the memorability scores using either measure, so the limits of agreement (LoA) are valid (see S4 Fig Taken together with the high rank correlation between the adolescent and adult memorability scores, we can conclude that memorability is highly consistent and reliable across these two age groups.

Within the realm of adolescent cognition, the significance of visual memorability extends notably to educational contexts, particularly concerning individuals in their youth who contend with specific developmental or learning disorders. The present investigation has revealed a notable degree of consistency in memorability ratings derived from cohorts of adolescents and adults. This discovery underscores the inherent characteristic of image memorability and substantiates the viability of utilizing large image memorability datasets, curated through the participation of adult subjects, for prospective inquiries involving the adolescent demographic.

A notable challenge inherent to online studies, including ours, is verifying participants’ self-reported ages. Given the nature of online experiments, there’s no foolproof way to confirm the accuracy of age information provided by participants. This limitation is not unique to our study but is a common concern in online research settings. Future studies might consider conducting in-person experiments, where age verification is more feasible. Approaches like recruiting participants through schools, sports teams, and youth clubs can also help mitigate the risk of inaccurate age reporting typically associated with online methodologies.

## Supporting information

S1 Fig Box-plot depicting distribution of collected memorability scores in younger adolescents, older adolescents, and adults, per the (*H* − *F*)/*N*_*resp*_ measure. Adult data is from the MemCat dataset [11]. The dashed horizontal lines represent the global mean memorability score for each of the populations (*M* = 0.74, *M* = 0.74, and *M* = 0.76 for younger adolescents, older adolescents, and adults, respectively). *n* = 1, 000 for all figures.
(TIF)

S2 Fig Memorability scores per image in adolescent and adult populations, per the (*H* − *F*)/*N*_*resp*_ measure. Memorability scores per image in adolescent and adult populations, per the (*H* − *F*)/*N*_*resp*_ measure. Each dot represents one image. The diagonal line represents identical memorability scores in adolescents and adults. Points are equally distributed around the line for all categories. Rank correlation is highest in the Sport category (C) and lowest in the vehicle category (E).
(TIF)

S3 Fig Down-sampled memorability scores per image in adolescent and adult populations, Per the (*H* − *F*)/*N*_*resp*_ measure. Estimates are based on 25 random splits. *N*_*resp*_ refers to the number of data points per age group used for the given calculation (to the total number of data points per image). Images were matched to equal number of responses in both the adolescent and adult age groups, and down-sampled to further explore matched correlations at different levels. Adolescent and adults memorability scores are indeed very similar, even when accounting for the slight differences in number of responses in the two groups.
(TIF)

S4 Fig Bland-Altman plot of Memoir and MemCat memorability scores. Assesses if there is any relationship between the mean memorability scores and the discrepancies between the two age groups. The red lines represent the mean difference between the adolescent and adult memorability scores using the (*H*/*N*_*resp*_ and (*H* − *F*)/*N*_*resp*_ memorability scores (0.02 and 0.09, respectively). The gray lines represent the 95% limits of agreement.
(TIF)

S5 Fig Bland-Altman plot of Memoir and MemCat false alarm rates. Assesses if there is any relationship between the mean false alarm ratios and the discrepancies between the two age groups. The red line represents the mean difference between the adolescent and adult false alarm ratios (0.07). The gray line represents the 95% limits of agreement.
(TIF)

S6 Fig Box-plot depicting overall distribution of collected memorability scores in younger adolescents, older adolescents, and adults, per the *H*/*N*_*resp*_ measure. Adult data is from the MemCat dataset [11].
(TIF)

S7 Fig Box-plot depicting overall distribution of collected memorability scores in younger adolescents, older adolescents, and adults, per the (*H* − *F*)/*N*_*resp*_ measure. Adult data is from the MemCat dataset [11].
(TIF)
