# Supplementary figures and images for: Memoir study: Investigating image memorability across developmental stages
Source: PLoS One. 2023 Dec 20;18(12):e0295940. doi: 10.1371/journal.pone.0295940 (PMC10732434; doi:10.1371/journal.pone.0295940)

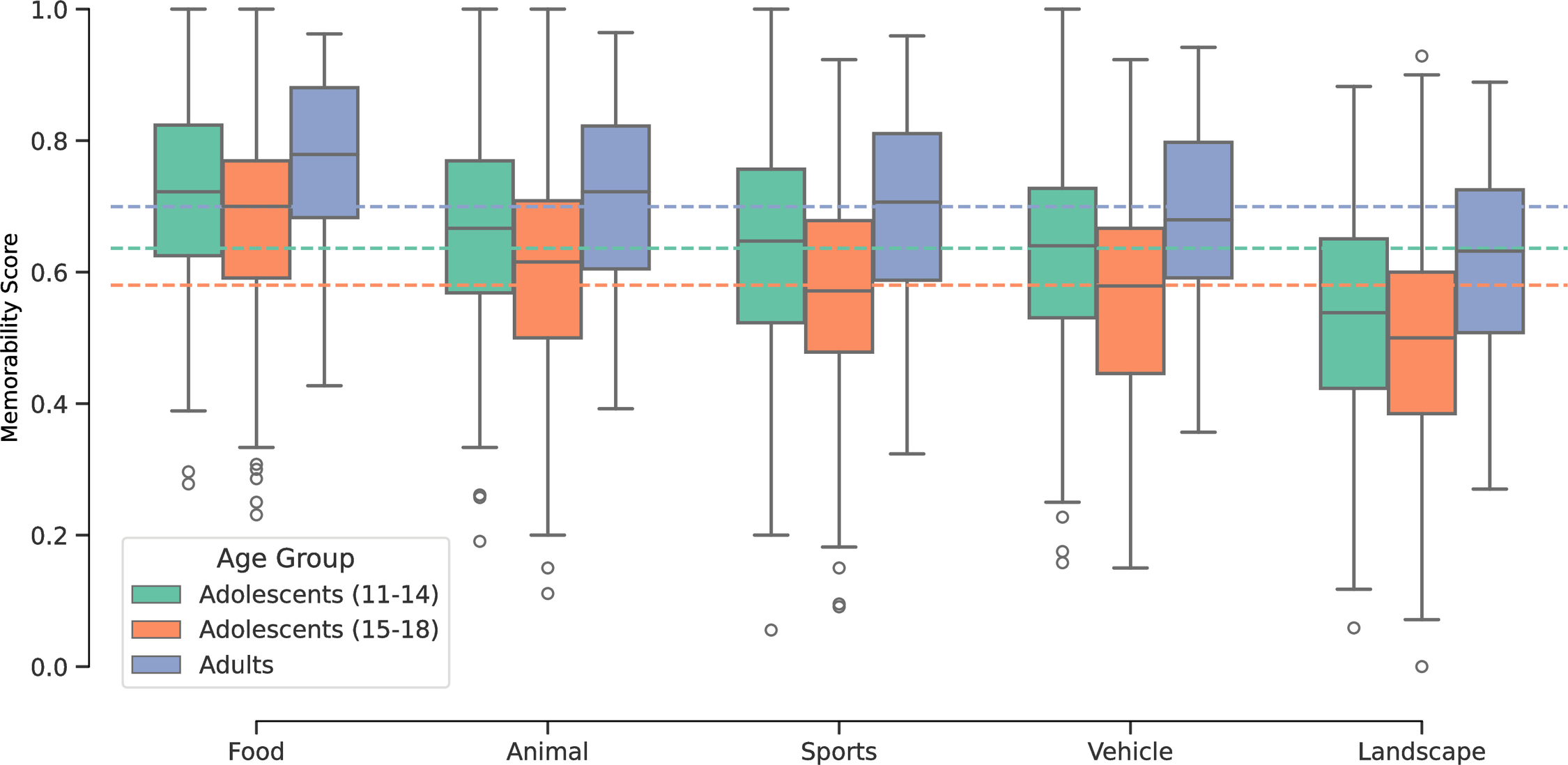

Supplement: S1 Fig — Adult data is from the MemCat dataset [11]. The dashed horizontal lines represent the global mean memorability score for each of the populations (M = 0.74, M = 0.74, and M = 0.76 for younger adolescents, older adolescents, and adults, respectively). n = 1, 000 for all figures. (TIF) [file pone.0295940.s001.tif]

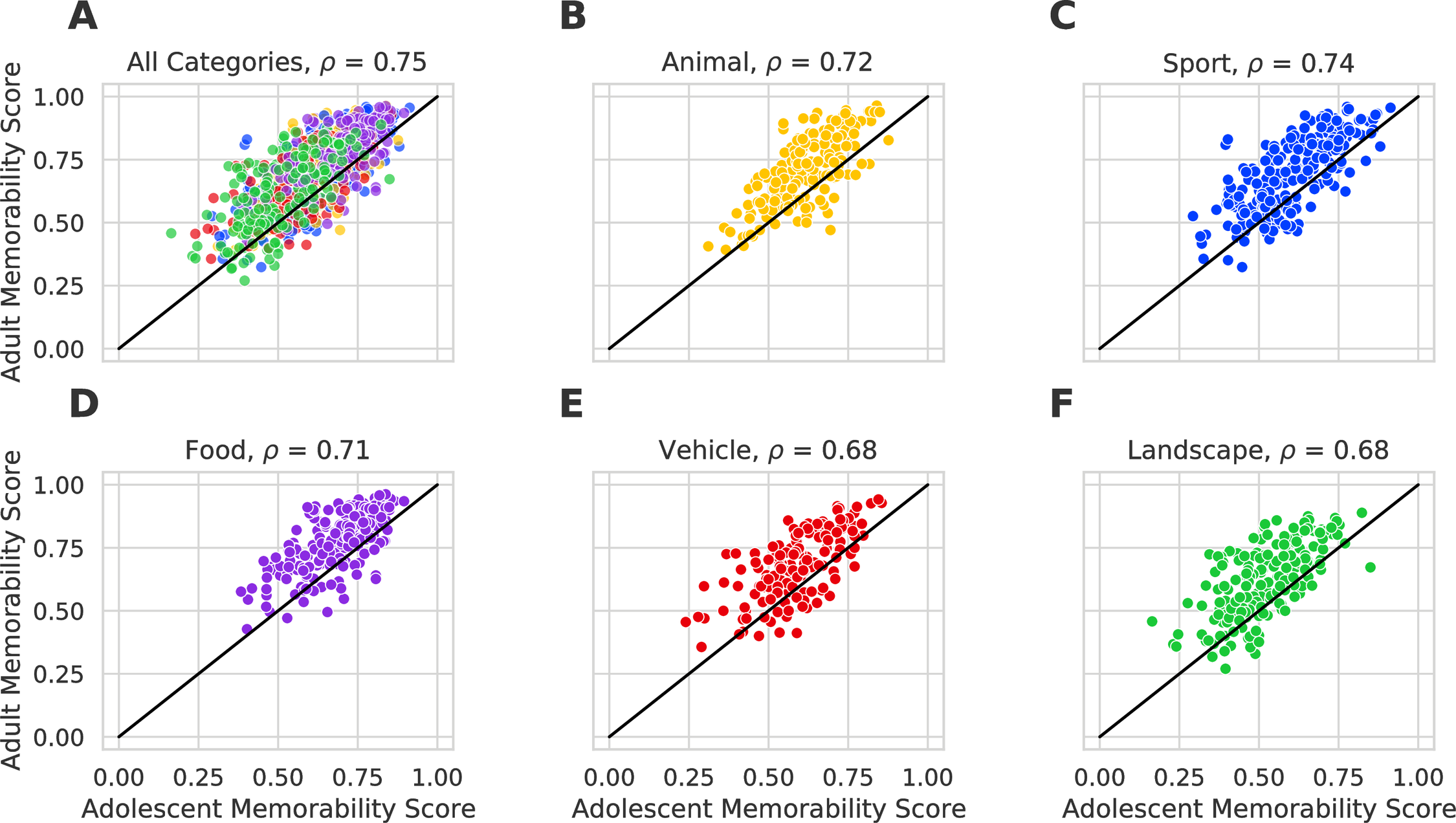

Supplement: S2 Fig — Memorability scores per image in adolescent and adult populations, per the (H − F)/Nresp measure. Each dot represents one image. The diagonal line represents identical memorability scores in adolescents and adults. Points are equally distributed around the line for all categories. Rank correlation is highest in the Sport category (C) and lowest in the vehicle category (E). (TIF) [file pone.0295940.s002.tif]

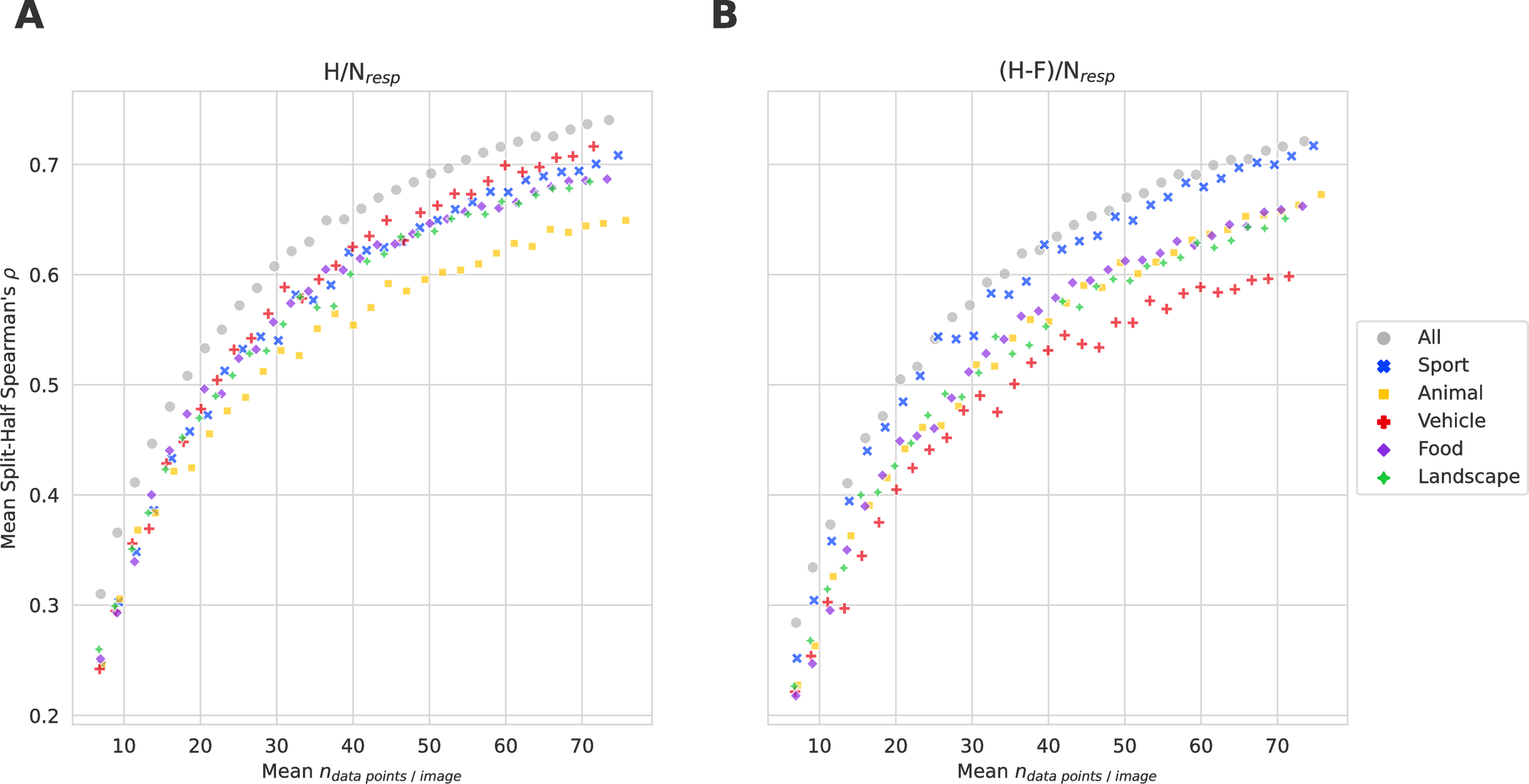

Supplement: S3 Fig — Estimates are based on 25 random splits. Nresp refers to the number of data points per age group used for the given calculation (to the total number of data points per image). Images were matched to equal number of responses in both the adolescent and adult age groups, and down-sampled to further explore matched correlations at different levels. Adolescent and adults memorability scores are indeed very similar, even when accounting for the slight differences in number of responses in the two groups. (TIF) [file pone.0295940.s003.tif]

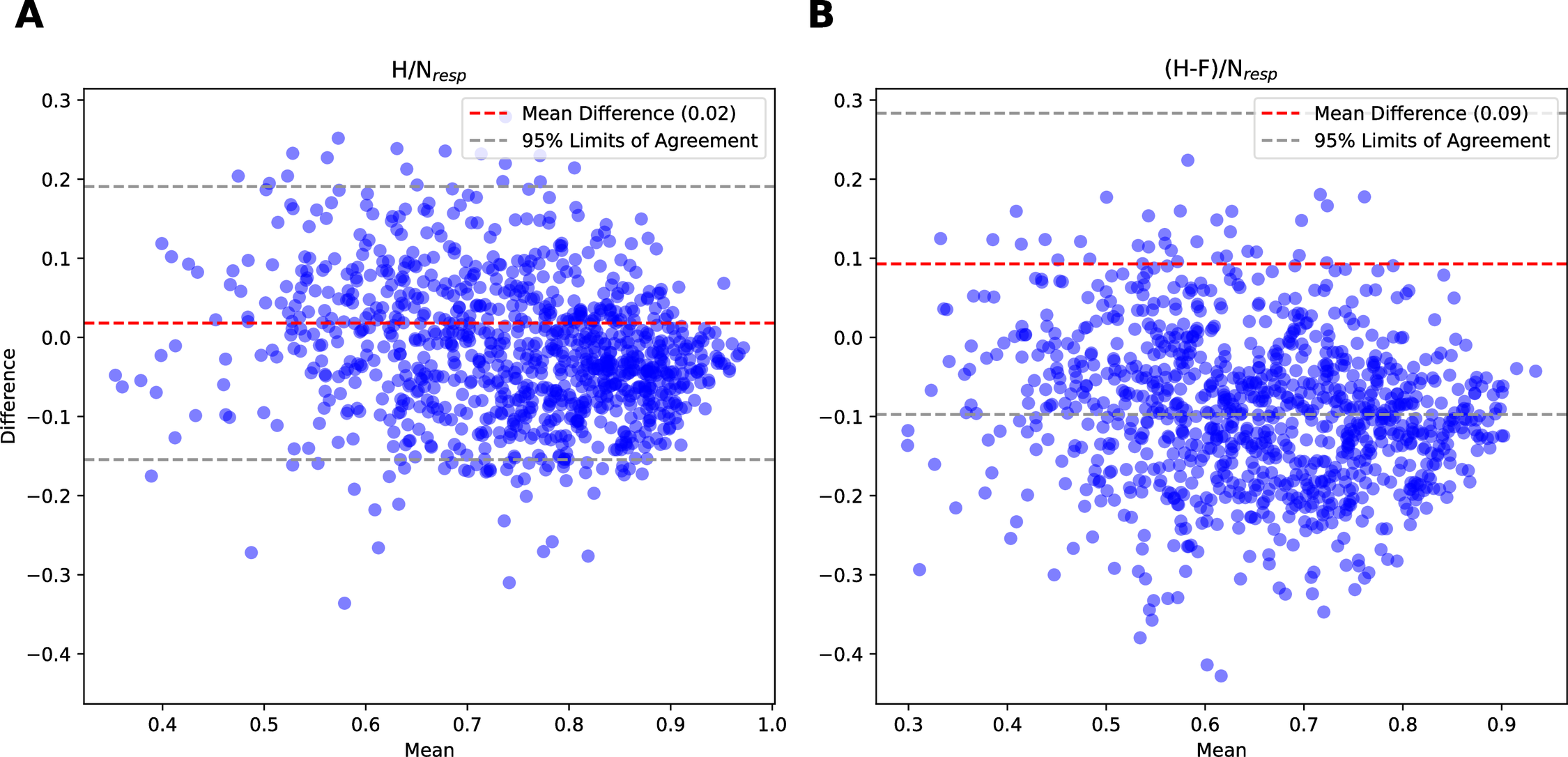

Supplement: S4 Fig — Assesses if there is any relationship between the mean memorability scores and the discrepancies between the two age groups. The red lines represent the mean difference between the adolescent and adult memorability scores using the (H/Nresp and (H − F)/Nresp memorability scores (0.02 and 0.09, respectively). The gray lines represent the 95% limits of agreement. (TIF) [file pone.0295940.s004.tif]

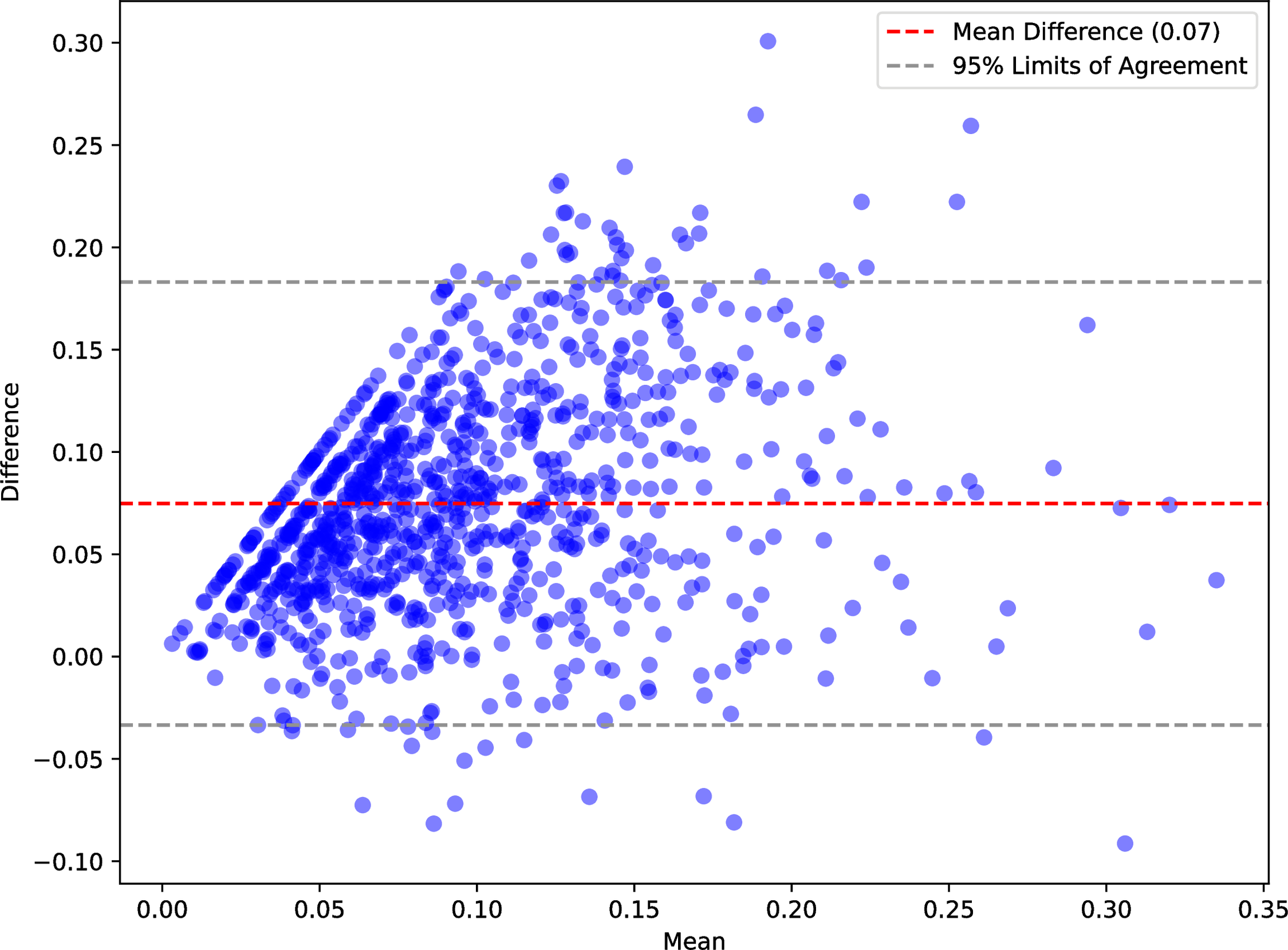

Supplement: S5 Fig — Assesses if there is any relationship between the mean false alarm ratios and the discrepancies between the two age groups. The red line represents the mean difference between the adolescent and adult false alarm ratios (0.07). The gray line represents the 95% limits of agreement. (TIF) [file pone.0295940.s005.tif]

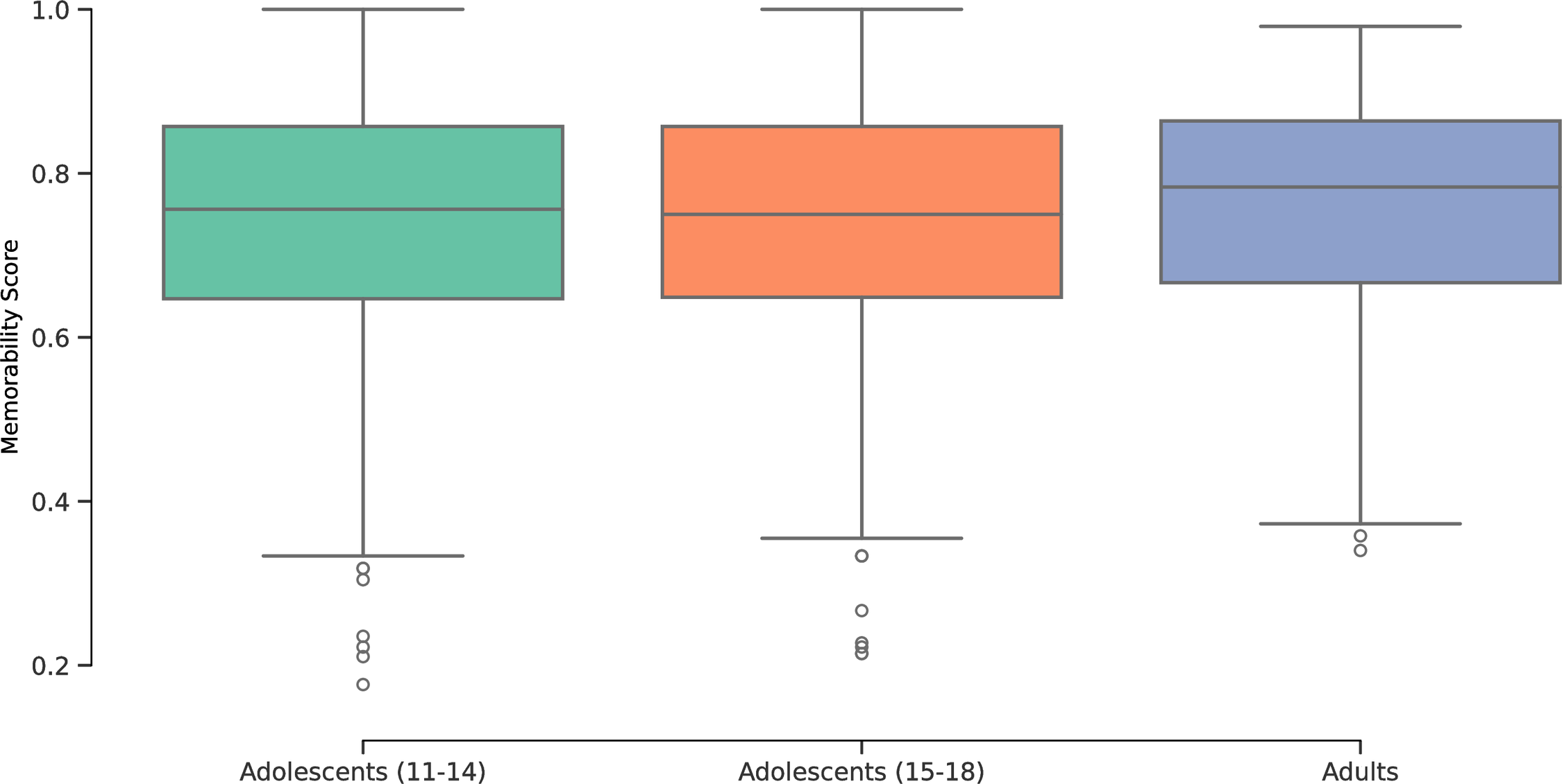

Supplement: S6 Fig — Adult data is from the MemCat dataset [11]. (TIF) [file pone.0295940.s006.tif]

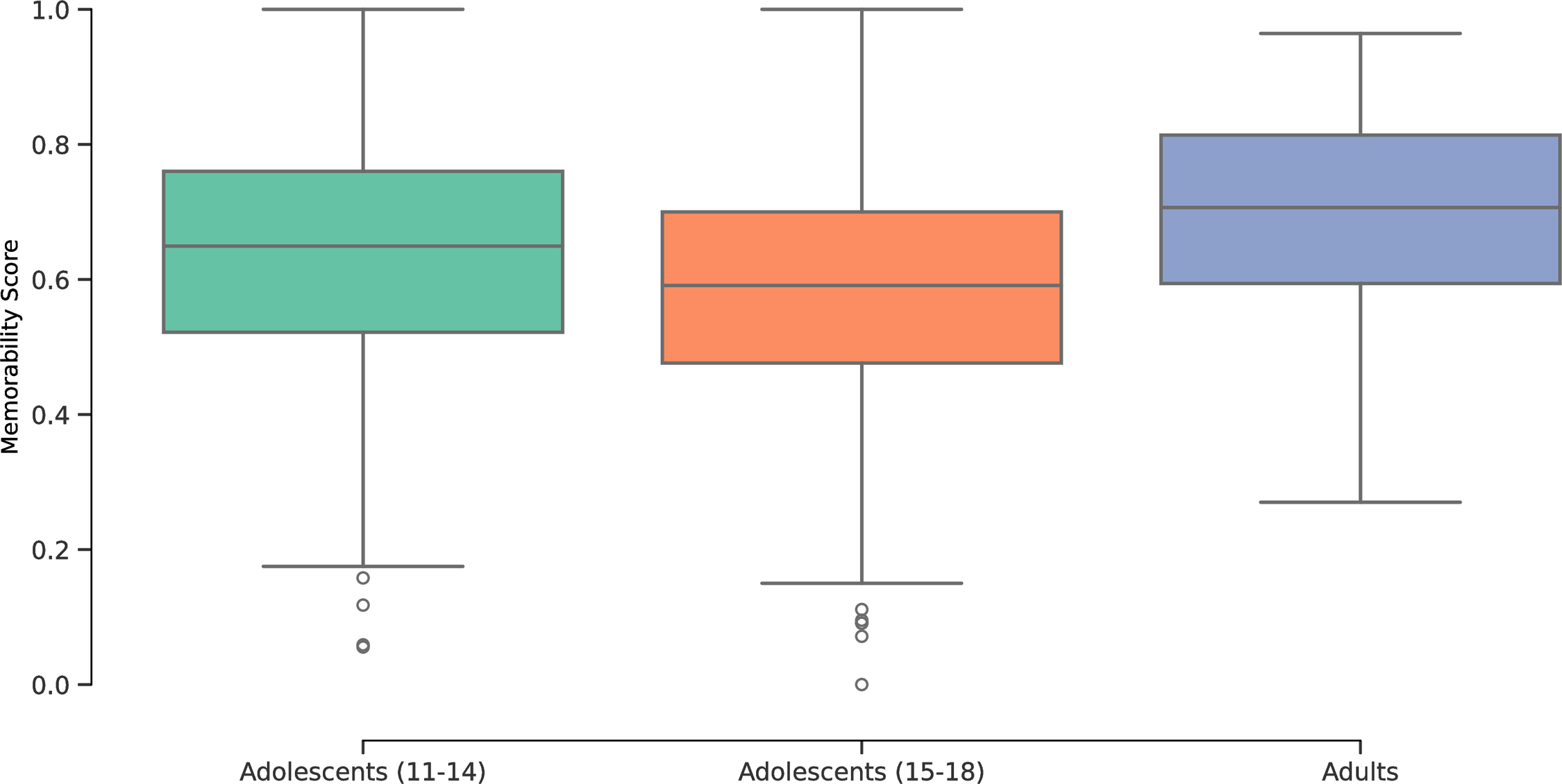

Supplement: S7 Fig — Adult data is from the MemCat dataset [11]. (TIF) [file pone.0295940.s007.tif]
